# [^68^Ga]pentixafor for CXCR4 imaging in a PC-3 prostate cancer xenograft model – comparison with [^18^F]FDG PET/CT, MRI and *ex vivo* receptor expression

**DOI:** 10.18632/oncotarget.21024

**Published:** 2017-09-16

**Authors:** Sarah M. Schwarzenböck, Jan Stenzel, Thomas Otto, Heike V. Helldorff, Carina Bergner, Jens Kurth, Stefan Polei, Tobias Lindner, Romina Rauer, Alexander Hohn, Oliver W. Hakenberg, Hans J. Wester, Brigitte Vollmar, Bernd J. Krause

**Affiliations:** ^1^ Department of Nuclear Medicine, Rostock University Medical Centre, 18057 Rostock, Germany; ^2^ Core Facility Small Animal Imaging, Rostock University Medical Centre, 18057 Rostock, Germany; ^3^ Department of Urology, Rostock University Medical Centre, 18057 Rostock, Germany; ^4^ Institute for Radiopharmaceutical Chemistry, Technische Universität München, 85748 Garching, Germany; ^5^ Institute for Experimental Surgery, Rostock University Medical Centre, 18057 Rostock, Germany

**Keywords:** prostate cancer, small animal PET/CT, [^68^Ga]Pentixafor, MRI, CXCR4

## Abstract

**Purpose:**

The aim was to characterize the properties of [^68^Ga]Pentixafor as tracer for prostate cancer imaging in a PC-3 prostate cancer xenograft mouse model and to investigate its correlation with [^18^F]FDG PET/CT, magnetic resonance imaging (MRI) and *ex vivo* analyses.

**Methods:**

Static [^68^Ga]Pentixafor and [^18^F]FDG PET as well as morphological/ diffusion weighted MRI and ^1^H MR spectroscopy was performed. Imaging data were correlated with *ex vivo* biodistribution and CXCR4 expression in PC-3 tumors (immunohistochemistry (IHC), mRNA analysis). Flow cytometry was performed for evaluation of localization of CXCR4 receptors (*in vitro* PC-3 cell experiments).

**Results:**

Tumor uptake of [^68^Ga]Pentixafor was significantly lower compared to [^18^F]FDG. *Ex vivo* CXCR4 mRNA expression of tumors was shown by PCR. Only faint tumor CXCR4 expression was shown by IHC (immuno reactive score of 3). Accordingly, flow cytometry of PC-3 cells revealed only a faint signal, cell membrane permeabilisation showed a slight signal increase. There was no significant correlation of [^68^Ga]Pentixafor tumor uptake and *ex vivo* receptor expression. Spectroscopy showed typical spectra of prostate cancer.

**Conclusion:**

PC-3 tumor uptake of [^68^Ga]Pentixafor was existent but lower compared to [^18^F]FDG. No significant correlation of *ex vivo* tumor CXCR4 receptor expression and [^68^Ga]Pentixafor tumor uptake was shown. CXCR4 receptor expression on the surface of PC-3 cells was existent but rather low possibly explaining the limited [^68^Ga]Pentixafor tumor uptake; receptor localization in the interior of PC-3 cells is presumable as shown by cell membrane permeabilisation. Further studies are necessary to define the role of [^68^Ga]Pentixafor in prostate cancer imaging.

## INTRODUCTION

For imaging of prostate cancer different imaging modalities have been evaluated in the last decades. Besides morphological imaging techniques, e.g. magnetic resonance imaging (MRI), functional as well as molecular imaging techniques such as positron emission tomography/computed tomography (PET/CT) are increasingly being used. PET radiopharmaceuticals addressing the prostate specific membrane antigen (PSMA) have already been integrated in the clinical routine. Recently, innovative and promising PET imaging biomarkers addressing new target structures such as chemokine receptors were developed. Chemokine receptors, e.g. CXCR4, are known to play a significant role in several entities of human cancers including prostate cancer - overexpression of CXCR4 is associated with tumor aggressiveness, progression, metastasis as well as poor prognosis [1–3]. [^68^Ga]Pentixafor targeting CXCR4 has successfully been evaluated preclinically and clinically in different malignancies such as brain tumors [4, 5], lung cancer [6, 7] as well as hematopoietic malignancies [8–10]. The aim of this study was to characterize the properties of [^68^Ga]Pentixafor as tracer for prostate cancer imaging in an aggressive PC-3 prostate cancer xenograft mouse model by small animal PET/CT. [^68^Ga]Pentixafor PET/CT data were compared to those of [^18^F]FDG PET/CT as well as morphological/functional MRI (tumor volume and apparent diffusion coefficient (ADC) values derived from diffusion weighted (DW) MRI and magnetic resonance spectroscopy (^1^H-MRS) data). Imaging data were compared with *ex vivo* biodistribution data of [^68^Ga]Pentixafor as well as with *ex vivo* CXCR4 expression of the tumors (assessed via immunohistochemistry (IHC) and mRNA analysis). Additionally, *in vitro* PC-3 cell experiments were performed using flow cytometry for evaluation of cell surface and internal expression of CXCR4 receptors.

## RESULTS

### [^68^Ga]Pentixafor and [^18^F]FDG small animal PET/CT

**Figure 1:**
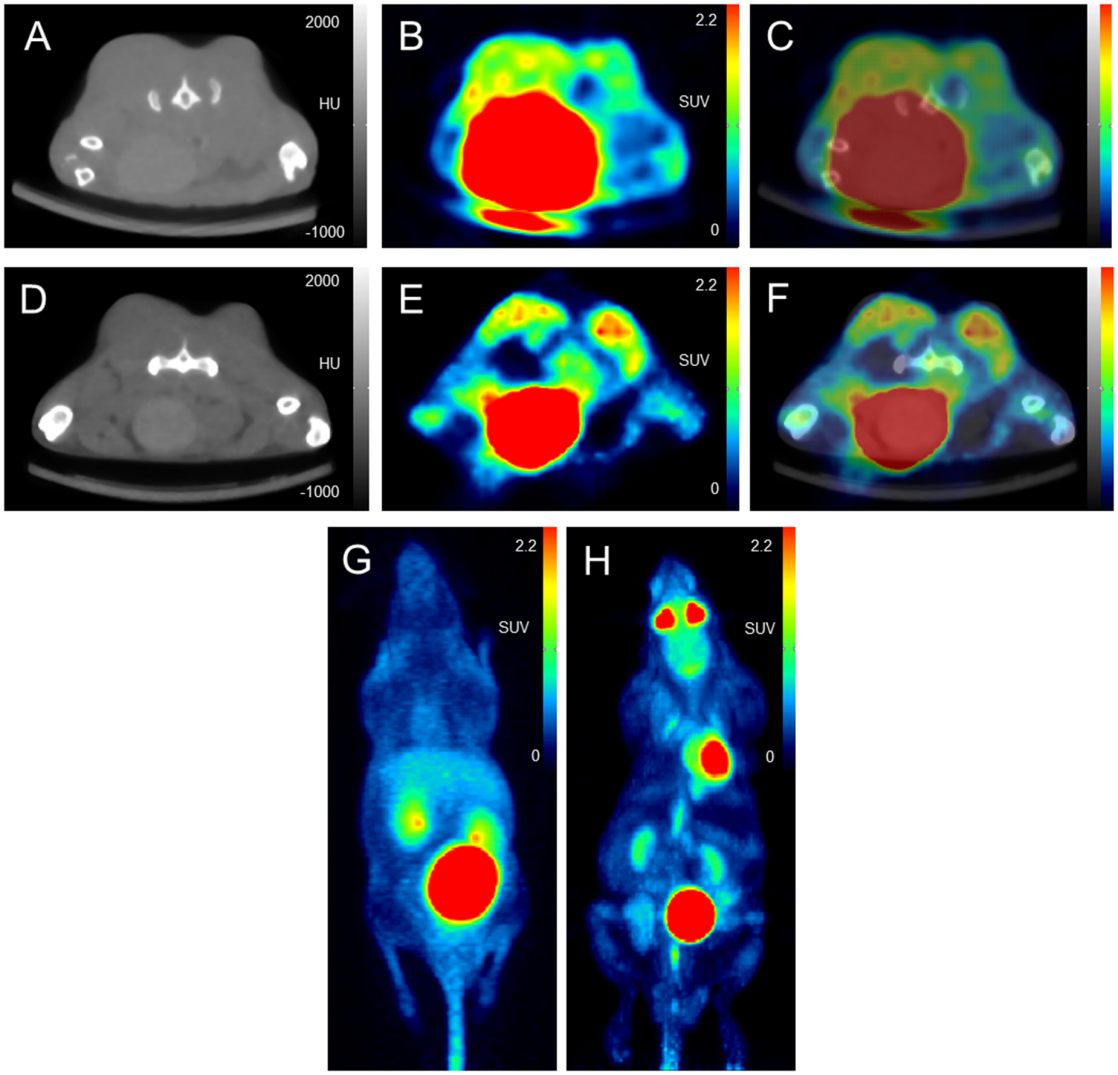
Image example: comparison of [^68^Ga]Pentixafor and [^18^F]FDG PET/CT in subcutaneous tumors (PC-3 cell line implanted in both flanks of a NMRI (nu/nu) mouse) Uptake of [^68^Ga]Pentixafor is lower compared to [^18^F]FDG. **(A-C)** [^68^Ga]Pentixafor (transaxial slices of (A) CT, (B) PET and (C) fused PET/CT); **(D-F)** [^18^F]FDG (transaxial slices of (D) CT, (E) PET and (F) fused PET/CT); maximum intensity projection (MIP) of [^68^Ga]Pentixafor **(G)** and [^18^F]FDG **(H)** showing different physiological tracer biodistribution of both tracers.

**Figure 2:**
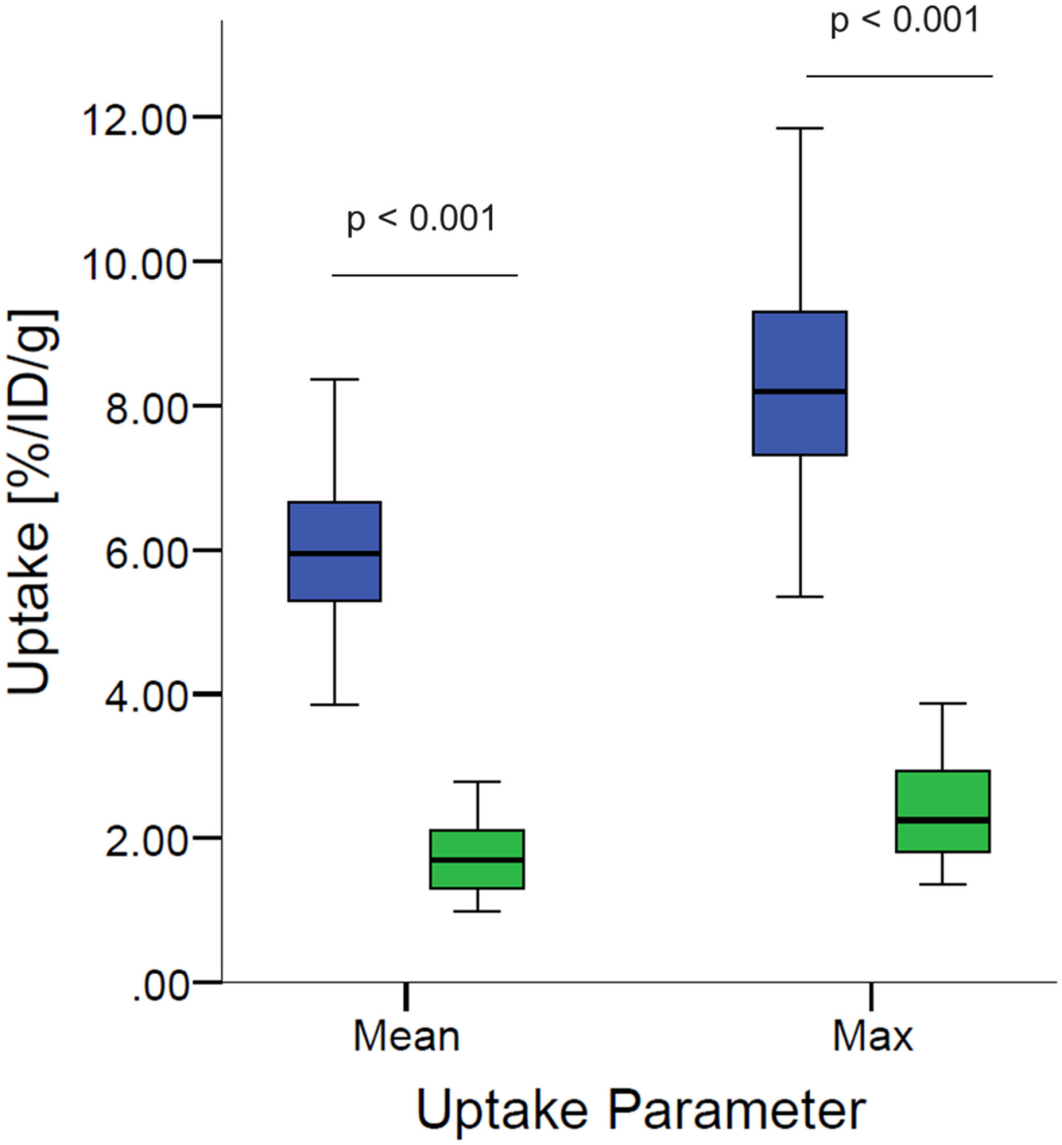
Boxplots of PET-derived mean %ID/g_mean_ and %ID/g_max_ of tumor tissue comparing [^68^Ga]Pentixafor (green) and [^18^F]FDG (blue) Tumor uptake of [^68^Ga]Pentixafor is significantly lower compared to [^18^F]FDG (p < 0.001, each).

**Figure 3:**
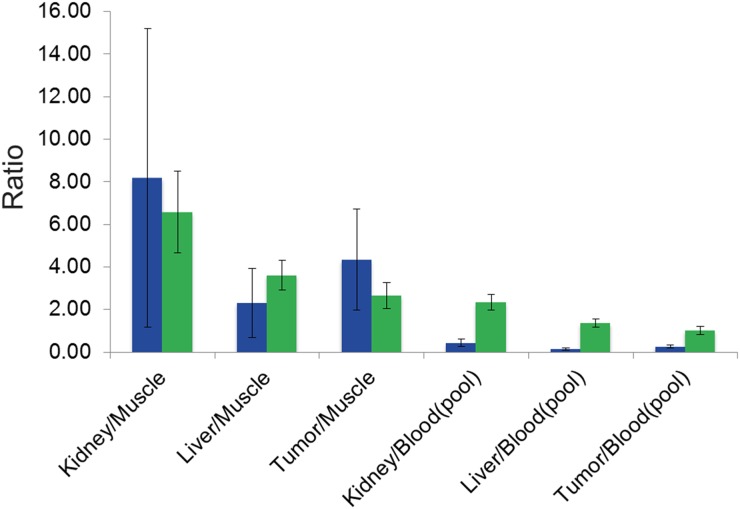
Diagram showing PET biodistribution of [^68^Ga]Pentixafor (green) and [^18^F]FDG (blue) with PET-derived mean K/M_PET_-, L/M_PET_-, T/M_PET_-, and K/B_PET_-, L/B_PET_ and T/B_PET_-ratios (including standard deviation)

[^68^Ga]Pentixafor PET visualized PC-3 tumors with moderate contrast, for an example see Figure 1. Tumor uptake of [^68^Ga]Pentixafor was significantly lower compared to [^18^F]FDG (mean percentage injected dose/gram (%ID/g)_mean_ 1.8 ± 0.6 and 5.8 ± 1.2, respectively, p < 0.001; mean %ID/g_max_ 2.5 ± 0.8 and 8.2 ± 1.8, respectively, p < 0.001), see Figure 2. No significant correlation was found between the tumor uptake of the two tracers (for %ID/g_mean_ r = 0.176, p = 0.17; for %ID/g_max_ r = 0.136, p = 0.23). There was a significant correlation between mean metabolic tumor volume of [^68^Ga]Pentixafor and [^18^F]FDG (r = 0.723, p < 0.001). For [^68^Ga]Pentixafor mean tumor/muscle (T/M)_PET_, kidney/muscle (K/M)_PET_, liver/muscle(L/M)_PET_ and tumor/blood (T/B)_PET_, kidney/blood (K/B)_PET_, liver/blood (L/B)_PET_ was 2.66 ± 0.61, 6.57 ± 1.92, 3.62 ± 0.69, 1.01 ± 0.18, 2.34 ± 0.36, 1.37 ± 0.18, respectively and 4.34 ± 2.37, 7.10 ± 5.45, 2.30 ± 1.62, 0.26 ± 0.08, 0.44 ± 0.18, 0.14 ± 0.06 for [^18^F]FDG (Figure 3).

### Morphological T2 MRI and DW MRI derived ADC values

Median MRI derived tumor volume was 373.5 mm^3^ (range of 27.30 – 897.60). Mean ADC_mean_ of the tumors was 1.01 ±0.05 × 10^-3^ mm^2^/s (range of 0.90 – 1.12 × 10^-3^).

**Figure 4:**
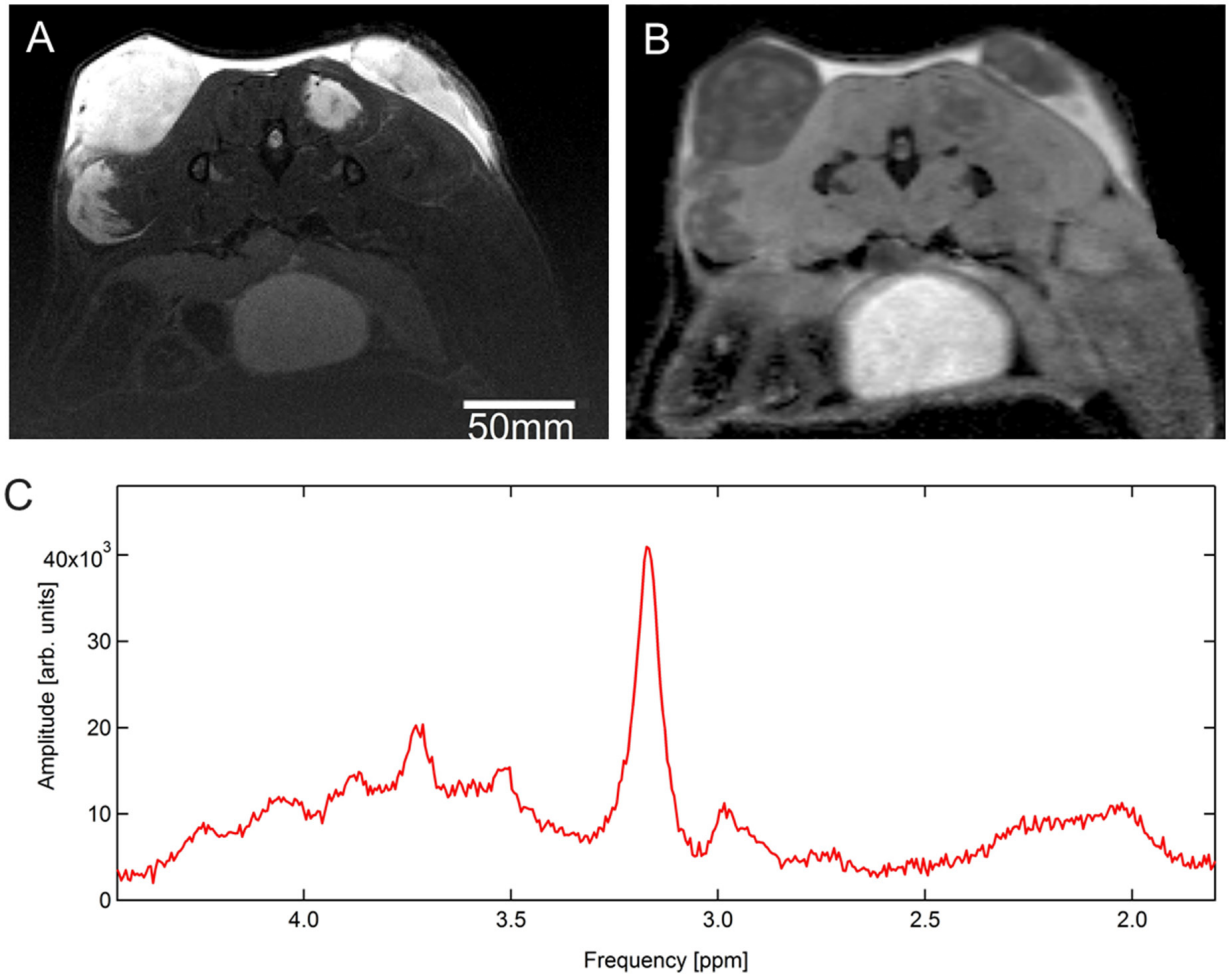
Image example: transaxial slices of **(A)** T2-weighted MRI and **(B)** DW MRI images of subcutaneous tumors (PC-3 cell line implanted in both flanks of a NMRI (nu/nu) mouse). In (A) tumor tissue appears hyperintense in the top part of the images whereas in (B) it appears hypointense. **(C)** Example of a tumor spectrum of one mouse with a prominent peak at 3.2 ppm representing choline and at 2.6 ppm representing citrate (^1^H-MRS pilot series).

For an example of T2 and DW MRI images see Figure 4A and 4B, respectively.

### ^1^H-MRS

In all 7 mice an increased choline peak could be shown, while only a very descrete or no citrate peak was observed. For an imaging example of a tumor spectrum see Figure 4C.

### *Ex vivo* biodistribution

**Figure 5:**
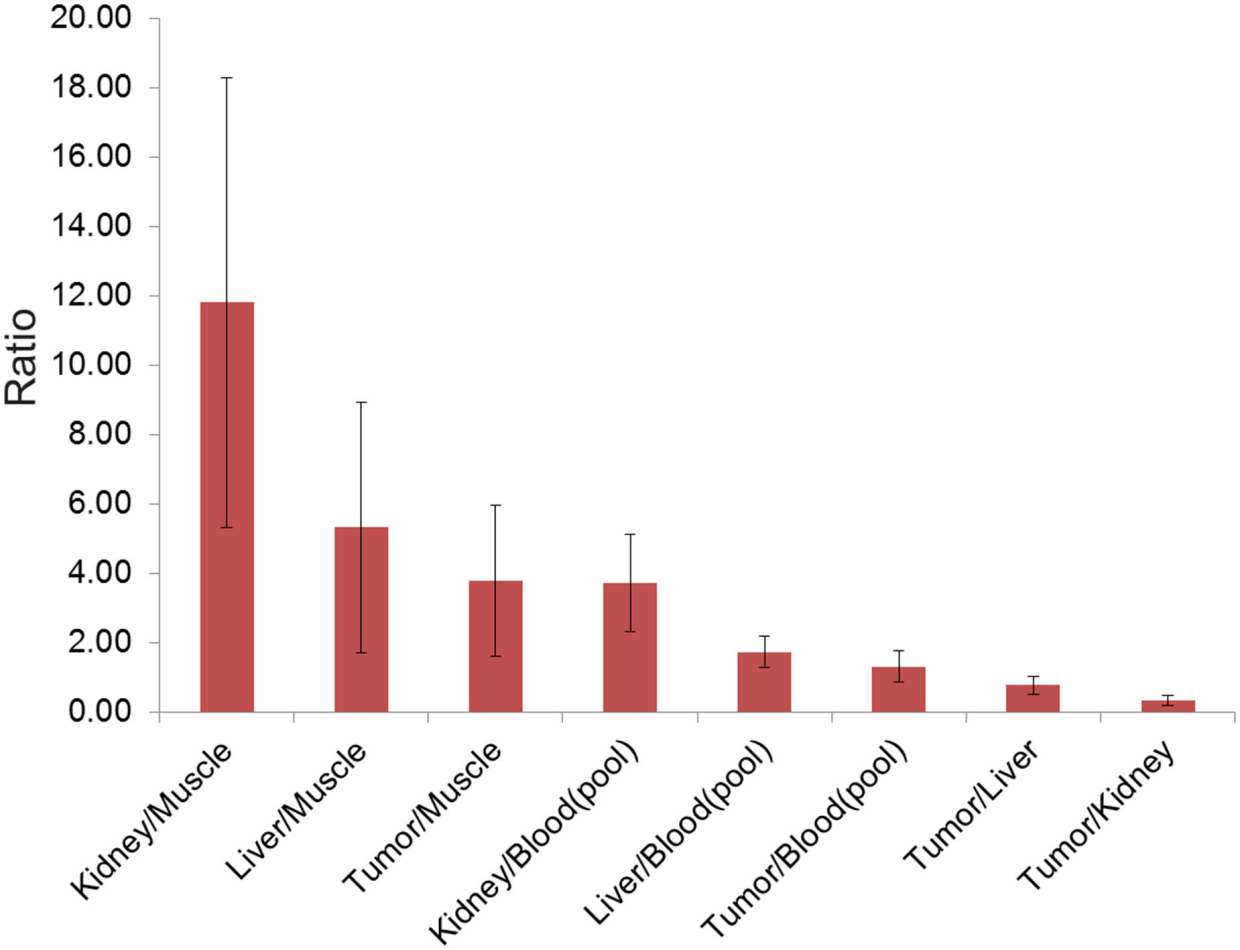
Diagram showing the results of *ex vivo* biodistribution (mean K/M_Bio_-, L/M_Bio_-, T/M_Bio_-, K/B_Bio_-, L/B_Bio_-, T/B_Bio_-, T/L_Bio_-, T/K_Bio_-ratio, including standard deviation) of [^68^Ga]Pentixafor with a high K/M_Bio_-, and L/M_Bio_-ratio due to high urinary excretion and liver metabolism

For [^68^Ga]Pentixafor mean tumor/muscle (T/M)_Bio_-, tumor/kidney (T/K)_Bio_-, tumor/liver (T/L)_Bio_-, tumor/blood (T/B)_Bio_-, liver/muscle (L/M)_Bio_-, kidney/muscle (K/M)_Bio_-, as well as liver/blood (L/B)_Bio_- and kidney/blood (K/B)_Bio_-ratio was 3.80 ± 2.20, 0.33 ± 0.15, 0.79 ± 0.26, 1.32 ± 0.46, 5.34 ± 3.62, 11.82 ± 6.50, 1.74 ± 0.45, 3.73 ± 1.41, respectively. Besides a moderate T/M_Bio_- and T/B_Bio_-ratio, a low T/K_Bio_- and T/L_Bio_-ratio was observed. L/B_Bio_- and K/B_Bio_-ratio was moderate, L/M_Bio_- and K/M_Bio_-ratio was high. For an overview see Figure 5.

### Immunohistochemistry for assessment of *ex vivo* CXCR4 expression in tumor tissue

**Figure 6:**
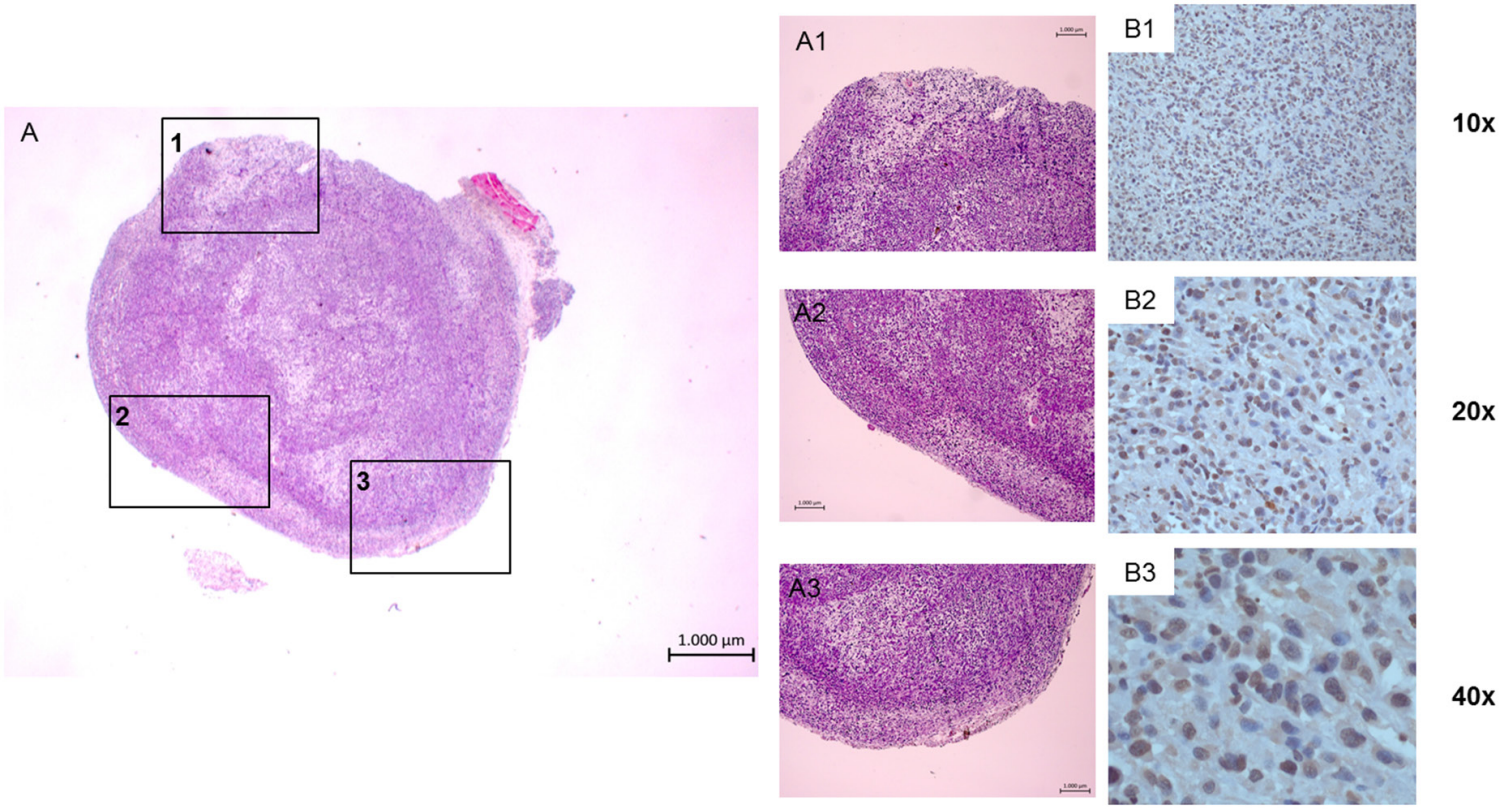
Image example of *ex vivo* tumor staining of a subcutaneous PC-3 tumor H.-E. staining: **(A)** overview of one transaxial slice, (A1-A3) amplification of three exemplary tumor regions. Labeling of CXCR4 positive cells using anti human CXCR4 antibody (brown), slices counterstained with hemalaun to visualize CXCR4 negative cells (blue) (B1-B3), different amplifications).

Expression of chemokine receptor subtype CXCR4 on tumor cells was demonstrated qualitatively and semiquantitatively by IHC (Figure 6). The mean percentage of CXCR4 positive cells was 60.91 ± 0.06, 60.27 ± 0.05 and 60.60 ± 0.05 for the whole tumor tissue, central part and peripheral part of the tumors indicating no relevant difference between those three tumor regions. Median immunoreactive score (IRS) was 3 [11].

### mRNA analysis for assessment of *ex vivo* CXCR4 mRNA expression in tumor tissue

**Figure 7:**
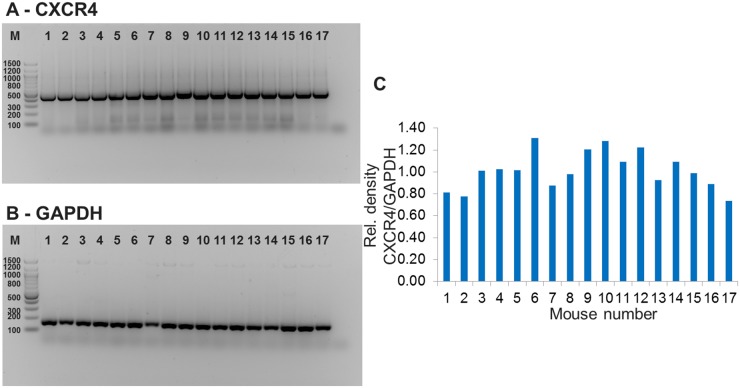
Results of mRNA analysis for assessment of *ex vivo* CXCR4 expression in tumor tissue Stained bands of PCR products of CXCR4 **(A)** and GAPDH **(B)**. Diagram showing relative mRNA expression of CXCR4 assessed densitometrically and normalized to GAPDH **(C)**.

Expression of CXCR4 mRNA expression in tumor tissue was demonstrated quantitatively and semiquantitatively via mRNA analysis. Mean relative mRNA expression of CXCR4 normalized to GAPDH expression was 1.02 ± 0.17 (range 0.74 – 1.31) (Figure 7).

### Correlation between [^68^Ga]Pentixafor uptake and CXCR4 receptor expression (IHC and mRNA analysis)

There was no significant correlation of [^68^Ga]Pentixafor uptake (%ID/g_mean_ and %ID/g_max_) and receptor expression assessed via IHC (p = 0.85, r = 0.05, each for the whole tumor) and mRNA expression analysis (p = 0.85, r = - 0.05 and p = 0.75, r = - 0.09, respectively).

### Flow cytometry for evaluation of cell surface and internal CXCR4 receptor expression

**Figure 8:**
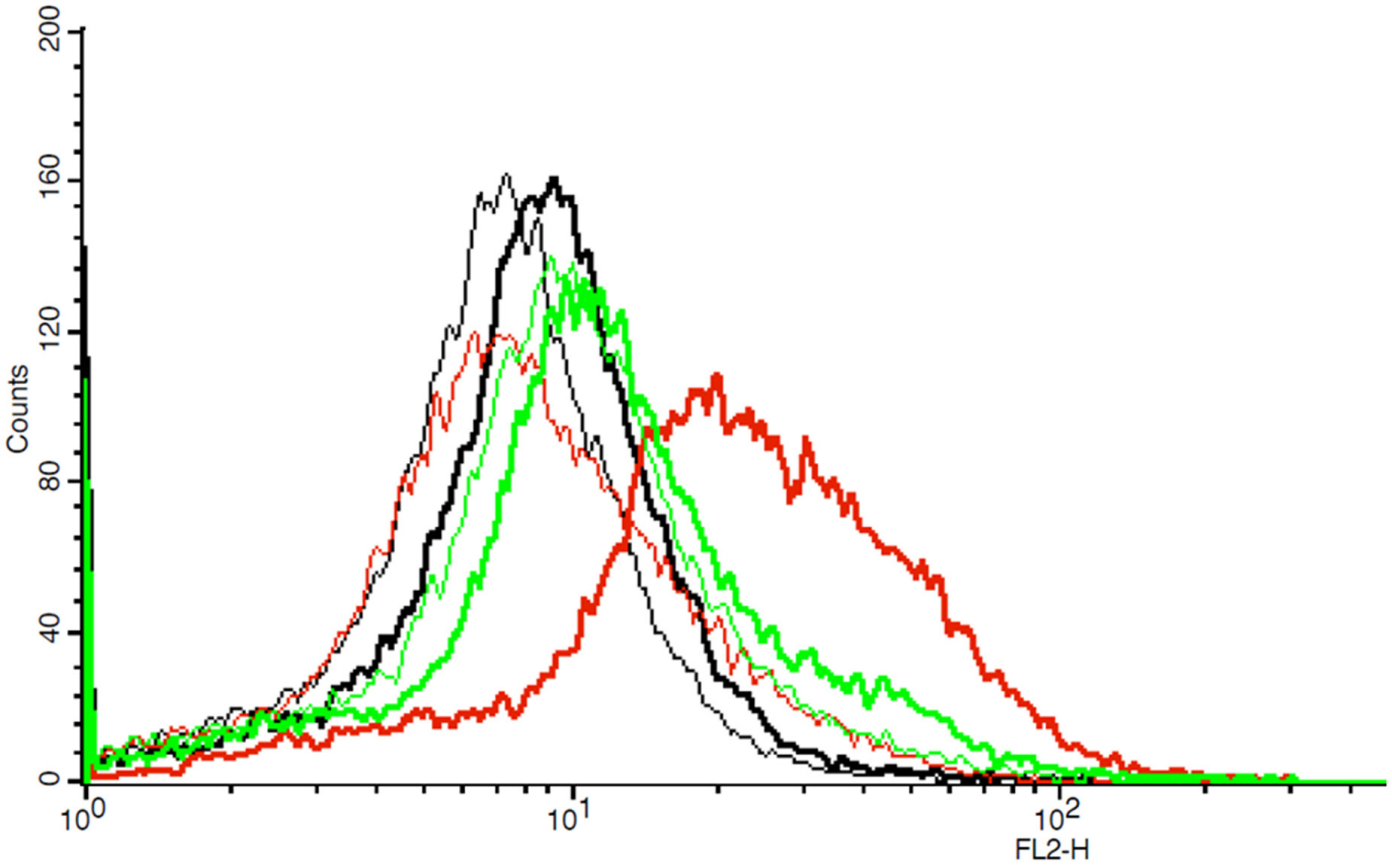
Example of one flow cytometric measurement showing graphs of fluorescence intensity (FL2-H) of IgG isotypic controls and CXCR4 in untreated, methanol-treated and TWEEN20-treated PC-3 cells, respectively (thin graphs: IgG isotypic controls, bold graphs: CXCR4; black: untreated cells, red: methanol-treated cells, green: TWEEN20-treated cells, respectively) Permeabilisation of cells shows a positive shift in fluorescence intensity.

In 3 independent measurements (each including double determination) untreated PC-3 cells showed an only slightly higher geographic mean fluorescence intensity (GMFI) for the anti-human CXCR4 antibody compared to the IgG isotypic control (Δ CXCR4 vs. IgG isotypic control 1.79 ± 0.45). Compared to IgG control as a reference, increase in CXCR4-positive cells was 4.12 ± 0.66% (Δ of positive cells CXCR4 vs. IgG-isotypic control). With methanol-permeabilisation the Δ mean GMFI in PC-3 increased up to 12.48 ± 3.8, while TWEEN20-treatment resulted in a lesser increase up to 3.96 ± 2.73 (Δ CXCR4 vs. IgG-isotypic control, each). These results were also reflected in the number of positive cells. Δ of positive cells CXCR4 vs. IgG-isotypic control increased up to 15.65 ± 3.04 % in methanol-permeabilised cells and up to 7.62 ± 1.75 % in TWEEN20-treated cells (for an example of a flow cytometric measurement see Figure 8). According to these results PC-3 cells seem to express only few CXCR4 on their cell surface; however permeabilisation (allowing for detection of CXCR4 located inside the cell) leads to a slight signal increase.

## DISCUSSION

In prostate cancer patients expression of chemokine receptors, such as CXCR4, is significantly associated with the presence of lymph node and bone metastasis and poor cancer-specific survival [1, 3]. Therefore PET/CT imaging targeting CXCR4 is a promising diagnostic approach potentially influencing future clinical management of advanced prostate cancer patients. According to the results of our study, tumor visualization in this PC-3 xenograft mouse model was feasible using [^68^Ga]Pentixafor. Tumor uptake was lower compared to [^18^F]FDG – this is not surprising as [^18^F]FDG is known to be highly uptaken by aggressive tumor cells. Our results on [^18^F]FDG tumor uptake are in line with previous studies on FDG PET/CT imaging in PC-3 prostate cancer xenograft mouse models [12, 13]. In accordance with our results, Vag et al. [14] found a lower uptake of [^68^Ga]Pentixafor compared to [^18^F]FDG PET in different solid cancers (including prostate cancer) in a small patient cohort. The authors supposed that detectability of solid cancers might be generally lower for [^68^Ga]Pentixafor PET compared to [^18^F]FDG PET; in hematopoietic malignancies the situation might be different as shown by Philipp-Abbrederis et al. [9].

Besides the higher tumor uptake of [^18^F]FDG, the physiological blood pool of [^18^F]FDG was much higher compared to [^68^Ga]Pentixafor. Both tracers are metabolized via the liver and excreted via the urinary tract. Liver uptake of [^68^Ga]Pentixafor is similar compared to [^18^F]FDG. Kidney uptake of [^68^Ga]Pentixafor was high, but still lower compared to [^18^F]FDG. Bladder activity of [^68^Ga]Pentixafor was high representing the predominant urinary excretion of the tracer [6].

The results of *in vivo* PET biodistribution of [^68^Ga]Pentixafor are in line with the *ex vivo* biodistribution data. Besides moderate T/M_Bio_- and T/B_Bio_-ratios, moderate L/B_Bio_- and K/B_Bio_-ratios and high L/M_Bio_- and K/M_Bio_-ratios were observed; in the consequence, tumor-to-organ ratios were low. These results are due to liver metabolism and high urinary excretion of [^68^Ga]Pentixafor as discussed above.

Besides PET/CT imaging morphological T2-weighted MRI allowed for accurate tumor visualization. DWI MRI derived mean ADC_mean_ value (1.01 ±0.05 × 10^-3^ mm^2^/s) was in line with results of human DWI MRI studies on prostate cancer [15–17]. MRS spectra obtained from our pilot series showed high choline peaks accompanied by reduced citrate peaks, therefore representing the typical spectral constellation of aggressive prostate cancer [18]. Given the small number of mice receiving MRS no quantitative assessment of MRS ratios was performed.

In mRNA analysis of PC-3 tumors the existence of mRNA of CXCR4 was demonstrated being in accordance with previous studies showing significantly upregulated mRNA levels of CXCR4 in PC-3 cells [19, 20]. In *ex vivo* IHC up to 60% of tumor cells showed CXCR4 expression, however, in semiquantitative evaluation IRS was rather low (median IRS of 3) indicating only a limited number of CXCR4 receptors on the cell surface of tumor tissue. This finding is in line with the limited *in vivo* [^68^Ga]Pentixafor PET signal (even though no significant correlations were found between CXCR4 expression shown *ex vivo* and *in vivo* by [^68^Ga]Pentixafor PET).

**Figure 9:**
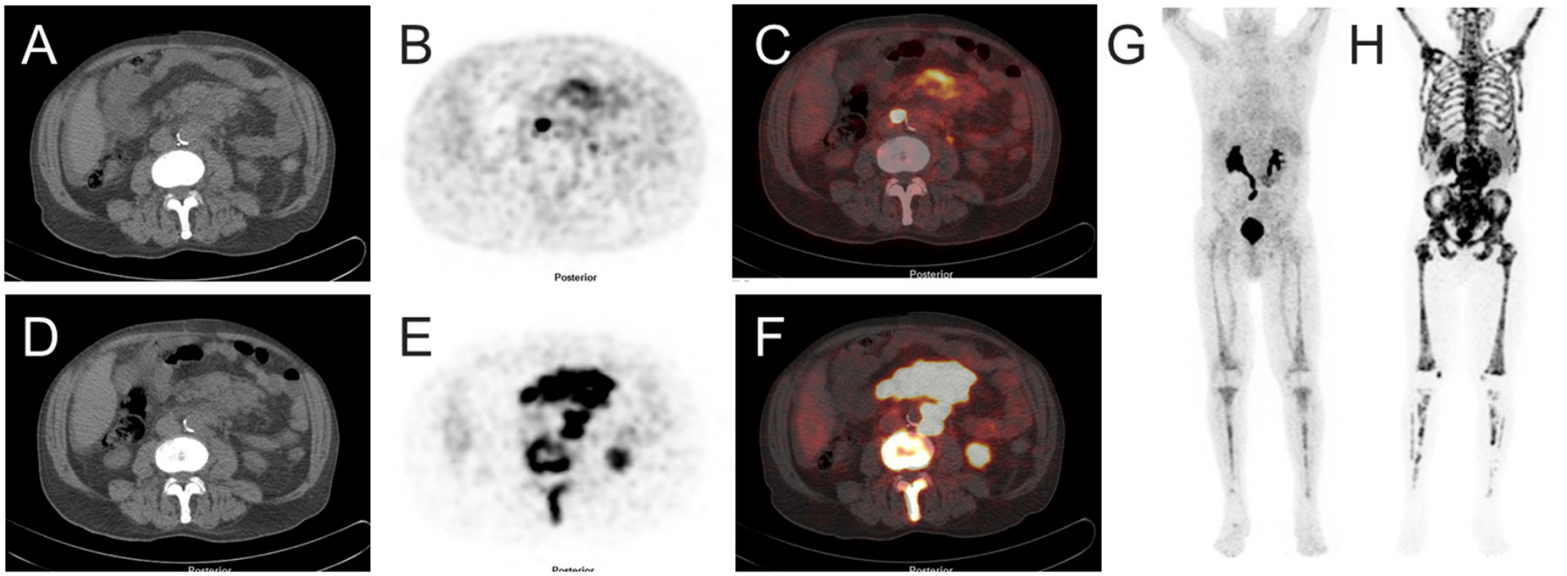
Image example of a clinically indicated [^68^Ga]Pentixafor PET/CT scan of a 74 year old patient with castration-resistant metastasized prostate cancer after all approved systemic therapies [^68^Ga]Pentixafor shows only faint or no increased uptake in disseminated nodal and bone metastases (whereas metastases are highly PSMA-positive on [^68^Ga]PSMA PET/CT) Transaxial sliced of **(A)** CT, **(B)** [^68^Ga]Pentixafor PET, **(C)** fused [^68^Ga]Pentixafor PET/CT and **(D)** CT, **(E)** [^68^Ga]PSMA PET, **(F)** fused [^68^Ga]PSMA PET/CT; maximum intensity projection of **(G)** [^68^Ga]Pentixafor PET and **(H)** [^68^Ga]PSMA PET.

Low *in vivo* uptake of [^68^Ga]Pentixafor was also observed in a clinical human PET/CT scan of a castration-resistant metastasized prostate cancer patient demonstrating only faint or even no uptake in the majority of highly PSMA-positive metastatic lesions (see Figure 9). As suggested by the low IRS, a possible explanation for the limited [^68^Ga]Pentixafor tumor uptake could be that CXCR4 might not always or only in parts be expressed on the cell surface (being a crucial prerequisite for successful PET imaging). Sun et al. hypothesized that CXCR4 expression in prostate cancer might be regulated at the protein level [21] – the influence of arrestin proteins on CXCR4 internalization and recycling has been shown by Cheng et al. [22]. To differentiate between CXCR4 receptors on the cell surface and internal CXCR4 receptors, flow cytometry was performed showing only faint GMFI for the anti-human CXCR4 antibody in a limited number of untreated PC-3 cells indicating low receptor expression on the cell surface; by cell membrane permeabilisation (enabling the antibody to bind to internally localized receptors), number of positive cells as well as PC-3 CXCR4 antibody GMFI increased. On the basis of these results it might be presumed that CXCR4 receptors are partially localized inside the PC-3 cells therefore not being accessible for [^68^Ga]Pentixafor.

The usefulness of [^68^Ga]Pentixafor PET/CT in prostate cancer imaging must specifically be addressed in future studies; once the use of [^68^Ga]Pentixafor PET/CT for prostate cancer imaging is more clearly defined it might potentially be used for monitoring of CXCR4 directed novel pharmacologic or endoradiotherapeutic therapeutic approaches in castration-resistant prostate cancer [23, 24].

### Conclusion

In this PC-3 prostate cancer xenograft model tumor visualization was feasible using [^68^Ga]Pentixafor PET/CT. However, [^68^Ga]Pentixafor tumor uptake was faint and lower compared to [^18^F]FDG uptake. No significant correlation of *ex vivo* CXCR4 receptor expression and [^68^Ga]Pentixafor uptake was shown. CXCR4 receptor expression on the surface of PC-3 cells was existent but rather low, being possibly due to internal receptor localization as shown by flow cytometric measurements. Further studies are necessary to define the role of [^68^Ga]Pentixafor in prostate cancer imaging and its use for patients` risk stratification or monitoring of CXCR4 directed therapeutic approaches.

### Limitations

For data analysis the measured activity concentration of each voxel was scaled to percentage injected dose/mass (%ID/g_mean_, %ID/g_max_) assuming a tissue density of 1g/cm^3^.

Due to performance characteristics of the PET system of the Inveon scanner, partial volume effects might have influenced the VOI values. To deal with the effects caused by spill-out and spill-in we used a threshold of 60% of the maximum value of an initial VOI to delineate the final tumor volume. Nevertheless, a potential effect of the limited spatial resolution of the scanner cannot be excluded. Also potential inter- and intra-observer variabilities should not be neglected as all of the VOIs have been placed manually.

## MATERIALS AND METHODS

### Prostate cancer tumor model

In this study an athymic human prostate cancer xenograft mouse model was used after subcutaneous implantation of androgen-independent, androgen receptor- and PSA-negative human PC-3 prostate cancer cells. The experimental protocol was approved by the local Animal Research Committee (Landesamt für Landwirtschaft, Lebensmittelsicherheit und Fischerei, Mecklenburg Vorpommern) (LALLF M-V/TSD/7221.3-1-051/14). Animal care was conducted according to the german legislation on protection of animals and the Guide for the Care and Use of Laboratory Animals (NIH publication 86-23 revised 1985). The PC-3 tumor was propagated in 5-7 weeks old, male athymic nu/nu mice (NMRI nu/nu Naval Medical Research Institute, Charles River Laboratories) (n=19, according to the results of biostatistical analysis) by subcutaneous injection of 1×10^7^ cells/injection suspended in 0.9% NaCl in a volume of approximately 100 μl per animal in both flanks without Matrigel. Palpable tumors developed within 2 to 4 weeks post implantation. In 2/19 mice only one tumor of one flank grew adequately.

For evaluation of tumor size all tumors were measured using a caliper (starting at day 7 after tumor cell implantation and then every 2 days until the time point of the first imaging. Initial median tumor volume at day 7 after tumor cell implantation was 8.4 mm^3^ (range 1.5 - 153.0 mm^3^) and at the time point of imaging 275.6 mm^3^ (range 72.2 – 873.0 mm^3^). Between those time points the tumors in the median grew by factor 22.6 (range 2.4 – 491.6). Median absolute change in tumor volume was 242.1 mm^3^ (range 48.2 – 871.3 mm^3^).

Mean time span between PC-3 tumor cell injection and start of imaging was 43.3 ± 6.2 days (range of 31-56 days).

### Synthesis of [^68^Ga]Pentixafor

[^68^Ga]Pentixafor was synthesized according to a previously described procedure [25, 6]. [^18^F]FDG was commercially purchased (Eckert & Ziegler, Berlin, Germany).

### PET/CT imaging protocol

A dual tracer small animal PET/CT study was conducted comparing [^68^Ga]Pentixafor and [^18^F]FDG. Mice were injected with 15 MBq [^68^Ga]Pentixafor and [^18^F]FDG, respectively, on two separate days via a microcatheter placed in a tail vein. Static PET/CT studies were performed in prone position 60 min p.i. using a commercially available preclinical PET/CT system (Inveon®, Siemens Healthcare Knoxville, USA). Whole body CT scans were acquired for attenuation correction and anatomical reference. Each PET data set was corrected for random coincidences, dead time and attenuation. However, no scatter correction was applied to avoid reconstruction artefacts possibly induced by the high activity concentration of the bladder due to the rapid renal clearance of both tracers, especially of [^68^Ga]Pentixafor. The 3D-PET imaging data were reconstructed with a 3D-ordered subset expectation maximization (OSEM)-algorithm (4 iterations, 6 subsets). Data were decay-corrected to the time of tracer injection. For details on the performance of the PET/CT system, imaging procedure and technical performance see Bao et al. and Kemp et al. [26, 27].

### Morphological and functional 7 T MRI imaging protocol

Animals were scanned in prone position using a 7 T small animal MRI (Bruker Biospec 70/30, 7.0 T; gradient inset: BGA-12S, 440 mT/m gradient strength) in combination with a transmit volume-resonator (86 mm inner diameter) and receive surface-coil (both: Bruker, Ettlingen, Germany).

The imaging protocol included morphological, transversal T2-weighted (T2w) RARE (Rapid Acquisition with Relaxation Enhancement) sequence with following parameters: TE/TR: 45/3800 ms; FoV: approx. 27 mm × 33 mm; matrix: 220 × 280; voxel size: 0.12 mm × 0.12 mm × 0.6 mm, approx. 50 slices.

In addition, DWI MRI was performed utilizing a DWI-spin-echo sequence [28, 29] with the following parameters: 5 b values (b = 0, 100, 300, 650, 1000 s/mm^2^), one A0 image; 3 directions; TE/TR: 22/2500 ms; matrix: 128 × 128; voxel size: 0.2 mm × 0.2 mm × 0.9 mm; min. 25 slices.

For ^1^H-MRS a triggered Point-Resolved Spectroscopy (PRESS) sequence with outer volume suppression and a voxel size of 2 mm x 4 mm x 4 mm (placed in the center of the tumor) was used: TE/TR: 32/2500 ms; 256 averages. The water signal was suppressed using the variable pulse power and optimized relaxation delays (VAPOR) scheme. Based on B_0_-field map measurements, the linewidth/spectral resolution was optimized by adjustments of first- and second-order shims.

### Mouse monitoring during imaging

Mice were anesthetized by inhalation anesthesia using isoflurane (volume of 1.2%-2.5%) and oxygen. Respiration was triggered during all MRI scans and monitored during all PET/CT scans (frequency: ~ 35-50 breaths/min). Temperature and respiration were controlled during the entire imaging period of all scans.

### Sequence of imaging

In 18/19 mice morphological and functional MRI was acquired first, followed by [^18^F]FDG PET/CT and [^68^Ga]Pentixafor PET/CT. Mean time range was 2.5 days between MRI and [^18^F]FDG PET/CT and 3 days between [^18^F]FDG and [^68^Ga]Pentixafor PET/CT. In 1/19 mice [^18^F]FDG PET/CT was acquired first, followed by MRI and [^68^Ga]Pentixafor one and 5 days later, respectively.

Sequence of PET/CT imaging was not randomized in order to allow for assessment of biodistribution of [^68^Ga]Pentixafor.

All 19 mice received morphological MRI. 18/19 mice received DW MRI, in one mouse DW MRI was not feasible due to technical reasons. 7/19 mice received ^1^H-MRS as a pilot series.

18/19 mice received [^18^F]FDG PET/CT (one mouse was lost due to death after MRI); 17/19 mice received [^68^Ga]Pentixafor PET/CT (one further mouse was lost due to death). After [^68^Ga]Pentixafor PET/CT all remaining 17 mice were euthanized following institutional regulations.

### [^68^Ga]Pentixafor and [^18^F]FDG small animal PET/CT - data analysis

PET data were analysed with PMOD 3.7 - analysis software (PMOD Technologies LLC, Zurich, Switzerland). Comparison of [^68^Ga]Pentixafor and [^18^F]FDG uptake was performed on static data sets. For each mouse, [^68^Ga]Pentixafor and [^18^F]FDG data analyses were carried out in the same manner. PET data were coregistered with CT data. Volumes of interest (VOI) were used to assess percentage injected dose/gram (%ID/g_mean_, %ID/g_max_) of tumor, muscle, liver, kidney and blood pool derived from attenuation–corrected PET emission data. For VOI placement CT images were used to delineate tumor contours, muscle tissue, kidneys on both sides, liver tissue as well as heart contour (for the calculation of blood pool activity).

VOIs were placed in tumor, muscle, liver, kidneys and heart in transaxial CT slices taking into account sagittal and coronal views for determination of organ/tissue borders. Two VOIs were placed surrounding the whole tumor tissue of both flanks. A threshold of 60% was used to ensure sole measurement of tumor tissue excluding necrotic parts. Two VOIs were placed in muscle tissue and in liver tissue each (representative VOIs of at least 0.3 × 0.3 × 0.3 cm, threshold 0%), in both kidneys (delineating the kidney contour on both sides using CT, threshold 0%), and heart (delineating the heart cavity using CT, threshold 0%) to assess blood pool activity.

All VOIs were transferred to the co-registered PET data, thresholds were applied as defined above. For statistical calculation we used VOI-derived %ID/g_mean_ and %ID/g_max_.

Tumor/muscle (T/M)_PET_-, kidney/muscle (K/M)_PET_-, liver/muscle (L/M)_PET_, tumor/blood (T/B)_PET_-, kidney/blood (K/B)_PET_-, and liver/blood (L/B)_PET_-ratios were calculated (defined as %ID/g_mean_ derived from tumor and organ VOIs, respectively, divided by %ID/g_mean_ derived from muscle and blood VOIs, respectively) for each mouse for [^68^Ga]Pentixafor and [^18^F]FDG.

### Morphological T2 MRI - data analysis

Morphological T2w images were analyzed employing Slicer3D software version 4.5 (National Institutes of Health, Bethesda, Maryland, USA). For volumetric measurement of the tumor multiple regions of interest (ROI) were drawn by hand following the outer surface of tumor tissue in each transaxial tumor slice. Afterwards these ROIs were used for creating VOIs.

### DW MRI - data analysis

DW MRI data were processed via vendor specific software (Image Display and Processing tool, Bruker Paravision 6.0.1). ADC-maps were calculated on a pixel by pixel basis from DW MRI data using a least square mono-exponential fitting [28]. To compute the mean ADC of the solid tumor tissue, hand drawn ROIs were placed in each transaxial tumor slice following the outer surface of tumor tissue. Afterwards these ROIs were used for creating VOIs.

### ^1^H-MRS - data analysis

^1^H-MRS data were evaluated using the jMRUI software package 5.2 [30, 31]. MRS spectra were fitted using the Hankel Lanczos singular value decomposition (HLSVD) algorithm [32]. Prior to fitting the spectra were phase-corrected (0^th^ order). For the fitting of metabolite peaks we identified the number of component peaks (typically 4) which should be contained in the spectral data. ^1^H-MRS data were analyzed qualitatively; a quantitative analysis taking into account metabolite ratios was not performed due to the small sample size.

### Post imaging procedures

After PET/CT imaging with [^68^Ga]Pentixafor, blood samples were taken from 17 mice by retrobulbar puncture (two mice were lost due to death after MRI and directly after [^68^Ga]Pentixafor injection, respectively). Immediately after this blood draw, all 17 animals were sacrificed by cervical dislocation under deep anesthesia following institutional regulations.

Tumors and organs (right kidney, liver and muscle) were collected. The larger one of both tumors of each mouse was fixed in total in 4% buffered paraformaldehyde (PFA) for paraffin-embedding and IHC. The other tumor was divided into two parts. One part was snap-frozen in liquid nitrogen and stored at -80°C for mRNA expression analysis, the other part was used for *ex vivo* biodistribution analysis. Tumor and organs of 17 mice were available for *ex vivo* analyses.

### *Ex vivo* biodistribution

For the biodistribution measurements of [^68^Ga]Pentixafor the amount of radioactivity from dissected organs (right kidney, liver and muscle) and tumor as well as blood samples were quantified as counts per minute per g tissue using a borehole (Captus® 700t, Capintec, Pittsburgh, USA) (median and mean time range between i.v. injection of [^68^Ga]Pentixafor and *ex vivo* measurement 130.1 and 127 ± 17.8 minutes, respectively; range 96 - 178 minutes). Tumor/muscle (T/M)_Bio_-, tumor/kidney (T/K)_Bio_-, tumor/liver (T/L)_Bio_-, tumor/blood (T/B)_Bio_-, liver/muscle (L/M)_Bio_-, kidney/muscle (K/M)_Bio_-, as well as liver/blood (L/B)_Bio_- and kidney/blood (K/B)_Bio_-ratios of biodistribution were calculated.

### Immunohistochemistry for assessment of *ex vivo* CXCR4 expression in tumor tissue

Tumors were post fixed at least 24 hours in buffered 4% para-formaldehyde. Tumors were then embedded in paraffin and 4 μm thick sections were cut and put on Polysine Adhesive Slides (Thermo Scientific). After deparaffinization with X-tra Slov (Medite), the antigen retrieval was performed in Target Retrieval Solution buffer pH 6.0 (Dako, Hamburg, Germany) for 7 min in a microwave. To stain for CXCR4, sections were treated with 5’peroxidase for 5 min and then incubated overnight at 4°C with monoclonal anti-human CXCR4 clone 44708 (1:200 R&D Systems). For detection of the CXCR4 antibody, the DAB chromogen Universal LSAB kit (System-HRP; DakoCytomation, Dako) was used according to manufacturer's instructions. The sections were counterstained with hemalaun, mounted with X-tra Kitt (Medite), visualized by light microscope (Olympus BX51, Hamburg, Germany) and digitally photographed with a Color View II FW camera (Color View, Munich, Germany).

The percentage of CXCR4 positive cells was visually quantified in 10 high power field (HPF) of 3 consecutive sections per tumor differentiating between whole tumor tissue, central part and peripheral part. Additionally, a semi-quantitative assessment was performed (calculation of IRS).

### Analysis of *ex vivo* CXCR4 mRNA expression in tumor tissue by semiquantitative PCR

Total RNA was extracted from 20-30 mg tumor tissue using the RNeasy Mini Kit (Qiagen, Hilden, Germany) according to the manufacturer's protocol. Afterwards cDNA was synthesized from 2 μg total RNA using SuperScript TM First Strand Synthese System (Invitrogen, USA). The PCR reaction mixture of CXCR4 as well as GAPDH contained 6,25 μL of 2x Kappa biosystem reaction buffer, 1 μg cDNA, 10 μM of each of forward and reverse primers in a final volume of 12,5 μL. PCR was performed as follows: first denaturation step at 90°C for 5 min and 30 cycles of 94°C for 15 s, 61°C for 30 s and 72°C for 10 s with a final extension at 72 °C for 10 min using a thermocycler (Eppendorf Mastercycler Gradient, New York, NY, USA). PCR products were run on 2% agarose gel containing 1xGelStar TM nucleic acid gel stain from Biotium (Hayward, CA, USA). Stained bands were visualized under UV light and photographed. The used primer sequences were CXCR4 forward primer: 5’-TCT TTG CCA ACG TCA GTG AG-3’; CxCR4 reverse primer: 5 ’TGG AGT GTG ACA GCT TGG AG-3’; GAPDH forward primer: 5’-ATC ACC ATC TTC CAG GAG CGA-3’; GAPDH reverse primer: 5’-GCC AGT GAG CTT CCC GTT CA-3. Signals were densitometrically assessed (Quantity One, ChemiDoc XRS System; Bio-Rad Laboratories, Munich, Germany) and normalized to GAPDH signals.

### Flow cytometry for evaluation of cell surface and internal CXCR4 receptor expression in PC-3 cells

To quantify and compare levels of CXCR4 receptor expression of the cell surface and internal CXCR4 expression of PC-3 cells flow cytometry was used with a phycoerythrin conjugated anti-human CD148 (CXCR4) antibody (BIOZOL, Eching, Germany, BLD-306506). Cells were harvested with trypsin-EDTA in medium (RPMI + 10% FCS + 1% Penicillin and Streptomycin) and centrifuged. Each sample (1 × 10^6^ cells) was washed once with PBS and incubated with 5 μl of the antibody solution for 40 minutes in darkness. The cells were washed with FACS-PBS (PBS + 0.5% BSA + 0.1% NaN_3_) and then analysed in 1 ml FACS-buffer-suspension using a Becton Dickinson FACSCalibur flow cytometer and analysed with Becton Dickinson CellQuest Pro.

To access internal receptors, a set of samples was permeabilised using 10 μl TWEEN20 in 1 ml of PBS for 30 minutes at 6°C followed by a twofold washing step with FACS-PBS, prior to adding the antibody. Subsequent steps remained equal to the unpermeabilised samples. Another set of samples was permeabilised using 3 ml 70% methanol instead of TWEEN20. The incubation dwell was 20 minutes at a temperature of -20°C. These measurements were independently repeated three times with double determination each.

### Statistical evaluation

Statistics were performed using Microsoft Excel 2008 and SPSS (version 20.0). Kolmogorov-Smirnov test revealed no normal distribution of data; therefore, statistics were performed using non-parametric tests. Wilcoxon signed-rank test for paired samples was applied to test for significant differences between uptake of [^68^Ga]Pentixafor and [^18^F]FDG (%ID/g_mean_ and %ID/g_max_). Correlations between the following data sets were evaluated using the Spearman rank correlation: uptake of [^68^Ga]Pentixafor and [^18^F]FDG (%ID/g_mean_ and %ID/g_max_), mean metabolic volume of [^68^Ga]Pentixafor and [^18^F]FDG, [^68^Ga]Pentixafor uptake (%ID/g_mean_ and %ID/g_max_) and CXCR4 expression assessed via IHC as well as via mRNA analysis, respectively. P-values less than 0.05 were considered statistically significant.

The Core Facility Multimodal Small Animal Imaging of the Rostock University Medical Center is funded by the *Deutsche Forschungsgemeinschaft* and EFRE (*Europäischer Fonds für regionale Entwicklung*).
